# Differential Expression of Metabolic Genes in Tumor and Stromal Components of Primary and Metastatic Loci in Pancreatic Adenocarcinoma

**DOI:** 10.1371/journal.pone.0032996

**Published:** 2012-03-07

**Authors:** Nina V. Chaika, Fang Yu, Vinee Purohit, Kamiya Mehla, Audrey J. Lazenby, Dominick DiMaio, Judy M. Anderson, Jen Jen Yeh, Keith R. Johnson, Michael A. Hollingsworth, Pankaj K. Singh

**Affiliations:** 1 The Eppley Institute for Cancer and Allied Diseases, University of Nebraska Medical Center, Omaha, Nebraska, United States of America; 2 Department of Biostatistics, University of Nebraska Medical Center Omaha, Nebraska, United States of America; 3 Department of Pathology and Microbiology, University of Nebraska Medical Center Omaha, Nebraska, United States of America; 4 Department of Surgery and Pharmacology, Lineberger Comprehensive Cancer Center, University of North Carolina at Chapel Hill, Chapel Hill, North Carolina, United States of America; 5 Department of Biochemistry and Molecular Biology, University of Nebraska Medical Center, Omaha, Nebraska, United States of America; 6 Department of Genetics, Cell Biology and Anatomy, University of Nebraska Medical Center, Omaha, Nebraska, United States of America; 7 College of Dentistry-Oral Biology, University of Nebraska Medical Center, Omaha, Nebraska, United States of America; Wayne State University School of Medicine, United States of America

## Abstract

**Background:**

Pancreatic cancer is the fourth leading cause of cancer related deaths in the United States with a five-year survival rate of 6%. It is characterized by extremely aggressive tumor growth rate and high incidence of metastasis. One of the most common and profound biochemical phenotypes of animal and human cancer cells is their ability to metabolize glucose at high rates, even under aerobic conditions. However, the contribution of metabolic interrelationships between tumor cells and cells of the surrounding microenvironment to the progression of cancer is not well understood. We evaluated differential expression of metabolic genes and, hence, metabolic pathways in primary tumor and metastases of patients with pancreatic adenocarcinoma.

**Methods and Findings:**

We analyzed the metabolic gene (those involved in glycolysis, tri-carboxylic acid pathway, pentose-phosphate pathway and fatty acid metabolism) expression profiles of primary and metastatic lesions from pancreatic cancer patients by gene expression arrays. We observed two principal results: genes that were upregulated in primary and most of the metastatic lesions; and genes that were upregulated only in specific metastatic lesions in a site-specific manner. Immunohistochemical (IHC) analyses of several metabolic gene products confirmed the gene expression patterns at the protein level. The IHC analyses also revealed differential tumor and stromal expression patterns of metabolic enzymes that were correlated with the metastasis sites.

**Conclusions:**

Here, we present the first comprehensive studies that establish differential metabolic status of tumor and stromal components and elevation of aerobic glycolysis gene expression in pancreatic cancer.

## Introduction

Cancer cells demonstrate significant alterations in energy metabolism in order to fuel uncontrolled cell proliferation and facilitate invasive properties [1]. Under aerobic conditions, normal cells utilize glucose to generate energy through glycolysis in the cytosol and Krebs cycle in the mitochondria [1]. However, as observed first by Otto Warburg, cancer cells reprogram glucose metabolism by facilitating the flux of glucose through glycolysis, while limiting the flux of glucose through Krebs cycle in mitochondria [2]. These alterations in the flux of glucose under aerobic conditions have been termed aerobic glycolysis [3]. Such reprogramming in energy metabolism significantly diminishes ATP production but facilitates rapid synthesis of metabolites, which generate building blocks to support rapid cell growth [4]. Decreased energy production during aerobic glycolysis is balanced by rapid glucose uptake that is accomplished in part by upregulation of glucose transporters such as GLUT1 [5]. Like most cancers, pancreatic ductal adenocarcinoma (PDAC) cells also demonstrate increased uptake of glucose [6]. This property of pancreatic cancer cells is exploited for noninvasive imaging of pancreatic tumors by utilizing a radiolabeled analog of glucose (18F-fluorodeoxyglucose, FDG) to visualize glucose uptake using positron emission tomography (PET) [7], [8].

Expression changes various metabolic pathway genes have been linked with clinical outcome in cancer patients [9]. Previous studies have linked expression alterations and polymorphisms in genes regulating glucose metabolism to clinical outcome in pancreatic cancer. A significant correlation has been identified between GLUT1 expression and pancreatic cancer prognosis [10]. Furthermore, higher expression of GLUT1 correlates with advanced stages of pancreatic intraepithelial neoplasias (PanIN) and non invasive intraductal papillary mucinous neoplasms (IPMN) [10]. Also, alterations in glucose metabolism have been linked with cell survival in pancreatic cancer [11]. In a large study, Dong *et al.* found that polymorphisms or mutations in glucose metabolic genes in patients with localized tumor, and in patients with advanced diseases were significant independent predictors for overall survival [12]. Thus, alterations in glycolytic enzyme expression and activities are critical to cancer prognosis.

Activating oncogene mutations and loss-of-function tumor suppressor mutations have been shown to further facilitate aerobic glycolysis. The oncogene Myc directly regulates expression of several glycolytic genes, and the oncogenes Ras and Src indirectly regulate the expression of glycolytic genes by regulating the expression of hypoxia inducible factor (HIF)-1, a key metabolic modulator [13]. The tumor suppressor p53 regulates expression of *TIGAR* (*T*P53-*i*nduced *g*lycolysis and *a*poptosis *r*egulator), which lowers the levels of fructose-2,6-bisphosphate in cells, resulting in an inhibition of glycolysis [14]. As p53 mutations are highly prevalent in pancreatic cancer, one effect is to relieve this glycolytic block and facilitate cancer progression. It has been hypothesized that most genetic alterations in tumors facilitate metabolic reprogramming, which, in turn, facilitates tumor growth and metastasis.

Although several studies have addressed changes in the expression levels of individual genes, the overall picture of metabolic reprogramming in pancreatic cancer remains far from clear [7], [10], [15]. In this study extensive expression analyses were evaluated to determine the expression levels of all the glucose metabolic genes in pancreatic tumors from primary and metastatic lesions. Several candidate metabolic genes, whose expression were significantly elevated in the primary tumors or in the tumors at individual metastatic sites in comparison to the normal tissues, were further evaluated for expression in tissue microarrays. The results clearly indicate elevation in the expression of several glycolytic genes in primary and metastatic tumors, suggesting a role of aerobic glycolysis in pancreatic cancer progression. Furthermore, we found that these genes were significantly overexpressed in tumor cells as compared to the stromal cells, suggesting that aerobic glycolysis may be more frequent in the tumor cell component of the pancreatic cancer.

## Methods

### Patients

PDAC samples from 35 patients with metastatic PDAC from the University of Nebraska Medical Center Rapid Autopsy Pancreatic Program (RAP) were evaluated for differentially expressed genes (at mRNA or protein levels). Human pancreatic tumors, metastatic lesions, and uninvolved specimens harvested by rapid autopsy from preconsented decedents previously diagnosed with pancreatic ductal carcinoma, were obtained from the University of Nebraska Medical Center's Tissue Bank through the Rapid Autopsy Pancreatic program. Written consent was obtained from patients prior to death. The Rapid Autopsy sample collection was performed as per the University of Nebraska Medical Center Institutional Review Board (UNMC-IRB) approval (approval # 091-01-FB).

### RNA Isolation and Microarray Hybridization

Frozen sections from each tissue sample (Table 1) were homogenized in TRIZOL reagent (Invitrogen Life Technologies, Carlsbad, CA USA). Total RNA was extracted using a standard chloroform protocol followed by purification with the Qiagen RNeasy Mini Kit (QIAGEN Inc, Valencia, CA USA). RNA integrity was evaluated by using RNA 6000 Nano LabChips on an Agilent 2100 Bioanalyzer (Agilent Technologies, Foster City, CA USA). RNA purity was assessed by the ratio of spectrophotometric absorbance at 260 and 280 nm (A260/280 nm) using NanoDrop ND-1000 (NanoDrop Inc, Wilmington, DE USA). All chips were prepared according to the manufacturer's instructions. Total RNA degradation was evaluated by reviewing the electropherograms and the RNA integrity number (RIN): only samples with preserved 18S and 28S peaks and RIN values greater than 7 were selected for gene expression analysis. One microgram of RNA was used as a template for DNA preparations and hybridized to Agilent 4×44 K whole human genome arrays (Agilent Technologies). cDNA was labeled with Cy5-dUTP and a reference control (Stratagene) was labeled with Cy3-dUTP using the Agilent (Agilent Technologies) low RNA input linear amplification kit and hybridized overnight at 65°C to Agilent 4×44 K whole human genome arrays (Agilent Technologies). Arrays were washed and scanned using an Agilent scanner (Agilent Technologies). All data is MIAME compliant and the raw data is publicly available in MIAME compliant Gene Expression Omnibus database (accession number: GSE21501).

**Table 1:** Tissue sections from pancreatic cancer patient autopsies that were utilized for gene expression arrays.

Subjects by tissue										
	Tissue									
Subject	Primary	Metastasis								
	Pancreas	Fat	Liver	Lung	Lymph node	Muscle	Bowel	Spleen	Vessel	Total
2	1	0	0	0	0	0	0	0	0	**1**
3	1	5	1	1	4	0	1	0	0	**13**
4	1	0	3	0	1	2	0	0	0	**7**
5	1	0	4	0	1	0	0	0	0	**6**
6	0	2	1	0	0	1	1	0	0	**5**
7	1	0	1	2	1	2	0	0	0	**7**
8	1	0	1	0	0	0	0	0	0	**2**
9	3	0	2	2	1	0	0	0	0	**8**
10	0	1	0	0	1	1	0	0	0	**3**
11	0	0	3	2	0	0	1	0	0	**6**
12	4	1	2	1	2	4	0	1	0	**15**
13	2	0	2	0	1	0	0	0	1	**6**
16	0	1	0	0	0	0	0	0	0	**1**
**Total**	**15**	**10**	**20**	**8**	**12**	**10**	**3**	**1**	**1**	**80**

### Tissue Microarray

The tissue microarrays (TMAs) are made from paraffin blocks of formalin fixed tissue from rapid autopsies. Large 2.0 and/or 2.5 mm cores are used to construct the tissue arrays. In addition to the tumor cores, each block contains control specimens of uninvolved kidney and colon tissue as well as pancreas from non-cancerous donors. Once completed, the tissue microarray blocks are cut into 4 micron thick sections and mounted on charged slides.

### Immunohistochemistry

Immunohistochemistry was performed by utilizing goat anti-rabbit IgG-AP with Fast-Red to produce red stain (*PicTure*™-Double Staining kit, Invitrogen), as per the manufacturer's instructions. Following primary antibodies were utilized: GLUT1 (Abcam, ab15309), HK2 (Cell Signaling Technology, 2867), Aldolase B (Genway, 20-203-421205), β-Enolase (Aviva Systems Biology, ARP48203_T10), PKM2 (Cell Signaling Technology, 3198), IDH3A (Abcam, ab58641). The intensity score was given by evaluating staining intensity of positive staining (0 = none; 1 = +weak; 2 = ++ intermediate, 3 = +++ strong). The proportion score representing the percentage of positively stained cell (0 = none; 1 = <5%; 2 = 5–25%; 3 = 26–50% 4 = 51–75% 5 = >75%). The overall protein expression in each sample is expressed as histoscore, which is the multiplication product of the proportion (0–5) and intensity scores (0–3) and is between 0–15, with a maximum of 15. The histoscore was calculated for the tumor cells and stromal cells separately. The staining score was evaluated by two independent observers and validated by a clinical pathologist.

### Statistical analysis

#### a. Microarray expression data

The two color Agilent arrays were used to quantify the relative gene expressions from the samples from either metastasis sites or primary cancer sites (labeled by red channel) and the reference human mRNA samples (Stratagene; labeled by green channel). The microarray expression data were analyzed to identify differentially expressed metabolic genes associated with certain metastasis site or primary pancreatic cancer. First all array data were preprocessed by filtering the data to eliminate poor quality spots or genes that did not have mean intensity greater than 10 for one of the two channels (green and red) in at least 70% of the experiments. The filtered data were normalized using the Lowess normalization. The log2 ratios between the intensity from red channel and green channel were calculated, and the ratios with at least 30 for the normalized raw intensity from both channels were included for further analysis. The analysis was conducted on 74 metabolic genes including 38 glycolytic genes, 25 TCA cycle genes, and 11 pentose phosphate pathway (PPP) associated genes (Table S1). The genes/enzymes involved in glucose metabolism were searched in Pubmed under ‘gene’ category (www.ncbi.nlm.nih.gov/gene) for all the potential isoforms of a given enzyme regulating the pathway. We noticed that a few genes were sampled more than once on the same array. The data from the same gene on the same array was then aggregated by taking an average. A mixed effect model involving fixed effect from the primary pancreatic cancer or metastasis site and random effect from subject was fit to each gene to account for the correlation among the samples from the same subject. The fixed effect from the primary pancreatic cancer or metastasis sites measures the difference in the log-scale expression between the primary cancer or metastasis and the reference mRNA samples. Estimates for the fixed effect and the p-value testing whether the fixed effect is equal to zero were calculated. Following that, the BH (Benjamini Hochberg) method [16] was used to control the false discovery rate (FDR) for multiple comparisons on all the selected metabolic genes. A gene was claimed to be differentially expressed only if the BH adjusted p-value is smaller than 0.05. Heatmaps were drawn on all 74 metabolic genes for relative mRNA levels for each site in each patient sample by utilizing the Genesis software [17]. The heatmap displayed the mean log2 fold change of the gene intensity between the samples from the metastasis site or the primary pancreatic cancer site and the reference mRNA samples in each patient. There are 8–20 samples per site. As we used the Agilent array, the Agilent Feature Processing for the experiments reported in this paper used the Feature Extraction Version 7.5.1 default settings recommended by Agilent. The SpotAnalyzer and the PolyOutlierFlagger algorithms define the feature areas and flag outliers. Spots are flagged and removed if pixel variation is too high or if the spot intensity is found to be an outlier. The genes were claimed to be differentially expressed for a certain metastasis site or for the primary pancreatic cancer site if p<0.05.

#### b. Tissue microarray data

The overall gene expression in each sample was quantified by a histoscore, which equals the product of the proportion score (at a scale of 0–5, representing the percentage of positively stained cell) and the intensity scores (at a scale of 0–3, evaluating staining intensity of positive staining). Median histoscore was reported for the stromal cells and tumor from different metastatic or pancreatic tumor sections on each gene. The histoscore values were compared between stromal cells and tumor cells from the same metastatic or pancreatic tumor section type on each gene using the nonparametric rank sum test. The Friedman's test was conducted on each gene to compare the histoscores between stromal cells and tumor cells after adjusting the site effects from different metastatic or tumor section.

## Results

### mRNA expression profile of glycolytic genes

**Figure 1:**
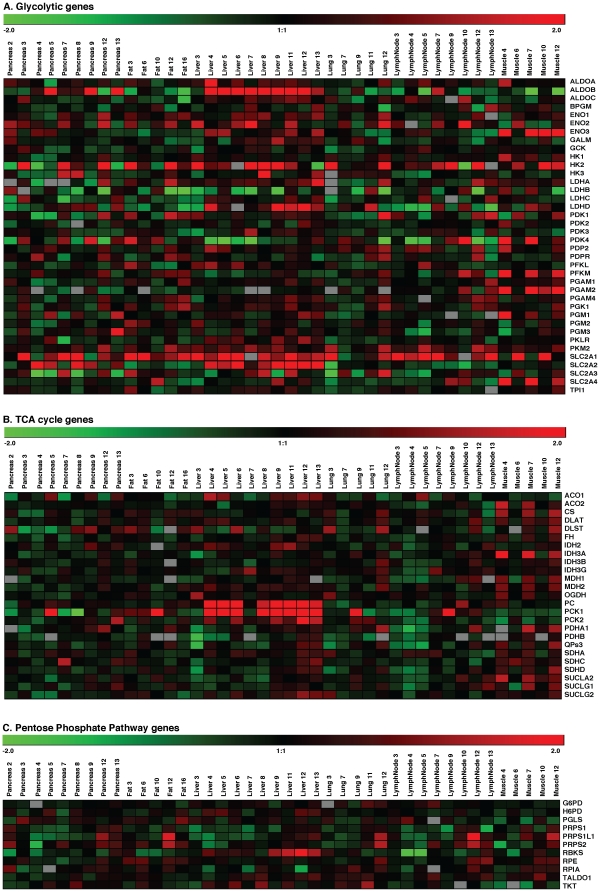
mRNA expression profiles of metabolic genes in primary and metastatic lesions. The mRNA expression profiles from pancreatic cancer patients were analyzed on 80 Agilent human whole genome 4×44 K DNA microarrays. The heatmap was created by Genesis [17]. The heatmap display the mean log2 fold change of the gene intensity between the samples from the metastasis site or the primary pancreatic cancer site and the reference mRNA samples. The genes examined were functionally involved in glycolysis (A), Krebs tricarboxylic acid cycle (B) or pentose phosphate pathways (C).

We evaluated primary and metastatic tumor lesions for alterations in metabolic gene expression signatures as compared to normal tissue by gene expression array analyses of RNA extracted from primary as well as metastatic tumor samples from pancreatic cancer patient autopsies (Table S1). Heatmap of glycolytic gene expression patterns in primary tumor or metastatic lesions relative to standard reference tissue are shown in Figure 1A. While we did not achieve stastistical significance for any genes in the primary pancreatic tumors and lung metastatic lesions, SLC2A1 (GLUT1) demonstrated robust overexpression in most other metastatic lesions (less than 0.05 FDR corrected p values and over 2 fold changes; Table S1). Similarly, PKM2 demonstrated modest but significant overexpression in most other metastatic lesions. HK2 was also overexpressed in fat and liver metastatic tumors. ENO3 was overexpressed in the liver and muscle metastasis sites. Liver and muscle metastatic lesions demonstrated significant upregulation of the highest number of glycolytic genes. A number of genes were expressed in a metastasis-site specific manner. SLC2A2 (GLUT2) and ALDOB were overexpressed in liver metastases, but had negative fold changes in the metastases at most of the other sites. SLC2A4 (GLUT4), PFKM and PGAM2 were highly expressed only in the muscle metastatic lesions compared to the reference mRNA.

### mRNA expression profiles of TCA cycle genes

Alterations in the expression of TCA cycle regulating genes were significant in liver or muscle metastasis sites. PC, ACO1, IDH2 and PCK1 and were overexpressed in tumor samples from liver metastases only (Figure 1B, Table S1). PC and PCK1 were the most upregulated (over 5-fold) genes in liver metastases. Several genes including CS, FH, ACO2, SUCLG1, SUCLA2, IDH3A, IDH3B, MDH1, PDHA1, and QPs3 were overexpressed only in muscle metastatic lesions. DLAT, and MDH2 were overexpressed in both liver and muscle metastatic lesions. While PCK2 was overexpressed in liver metastatic lesions, it was down-regulated in muscle metastases. Overall, most of the TCA cycle genes were upregulated in muscle metastatic lesions, indicating the role played by TCA cycle in metastatic colonization or tumor growth at the metastatic foci in skeletal muscles. We did not observe any enrichment of mRNA levels of TCA cycle associated genes in the primary pancreatic tumors. Most of the TCA cycle genes had negative fold changes in lymph node metastatic lesions, but the changes were not significant (FDR corrected p value>0.05).

### mRNA expression profiles of pentose phosphate pathway (PPP) associated genes

Most genes involved in the pentose phosphate pathway demonstrated only modest alterations in expression levels. However, ribulose-5-phosphate epimerase (RPE) demonstrated upregulation in fat and muscle metastasis sites (Figure 1C, Table S1). PGLS and TKT were downregulated in muscle metastases. PRPS1L1 and RPE were upregulated in muscle metastatic lesions, while RBKS was upregulated in liver. Glucose-6-phosphate dehydrogenase (G6PD), which catalyzes the first and the rate-limiting step in PPP, did not demonstrate significant alterations at mRNA levels.

### Immunohistochemical staining of differentially expressed genes

**Figure 2:**
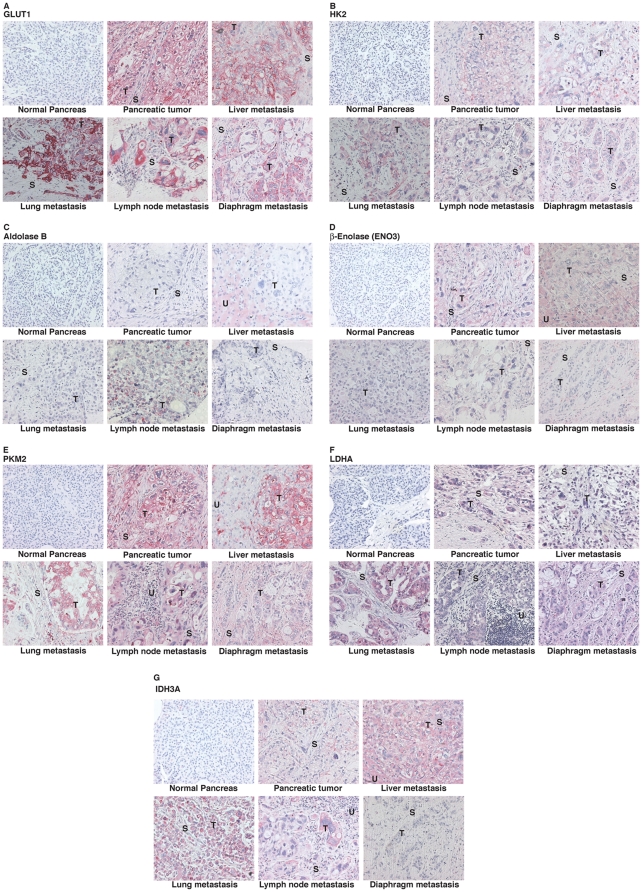
Metabolic protein expression in primary and metastatic lesions of pancreatic adenocarcinomas. Immunohistochemical examination of GLUT1 (A) HK2 (B), ALDOB (C), ENO3 (D), PKM2 (E), LDHA (F) IDH3A (G) expression in uninvolved pancreas, pancreatic tumor, and liver, lung, lymph node and diaphragm metastatic lesions. The staining was performed with Fast-Red stain. The images were captured at 200× magnification. Tumor cell area (T), stromal cell area (S) and uninvolved region (U) are indicated in the IHC images.

To confirm that alterations in the mRNA expression profiles of the metabolic genes were reflected in protein expression, we performed IHC staining for 7 metabolic genes on tissue microarrays representing uninvolved pancreas (normal), pancreatic tumor, and metastatic lesions from liver, lung, lymph nodes, and diaphragm. The metabolic genes selected were related to glucose uptake (SLC2A1 or GLUT1), glycolysis (HK2, ALDOB, ENO3, and PKM2), lactate production (LDHA) and TCA cycle (IDH3A). The genes were chosen to evaluate some robustly expressed genes from the microarray data sets (GLUT1, HK2); some modestly but consistently overexpressed gene (PKM2) and some genes overexpressed in a tissue specific manner (ALDOB and ENO3 for Muscle and liver, and LDHA for liver). Representative IHC images are illustrated in Figure 2. The images depicting the expression of each gene at multiple sites represent expression profiles in tissue samples from one pancreatic cancer patient. Hence, they may not be in perfect agreement with the median histoscore but still represent the inherent variability in expression. We observed minimal to no staining for these gene products in normal or uninvolved pancreas. All tissue sections were scored for expression in tumor and stroma. GLUT1 and HK2 were robustly expressed in most primary tumors and metastatic lesions. ENO3 and LDHA were moderately expressed. ALDOB expression levels were minimal in most tissues. IDH3A and PKM2 were significantly expressed in liver metastatic lesions.

**Figure 3:**
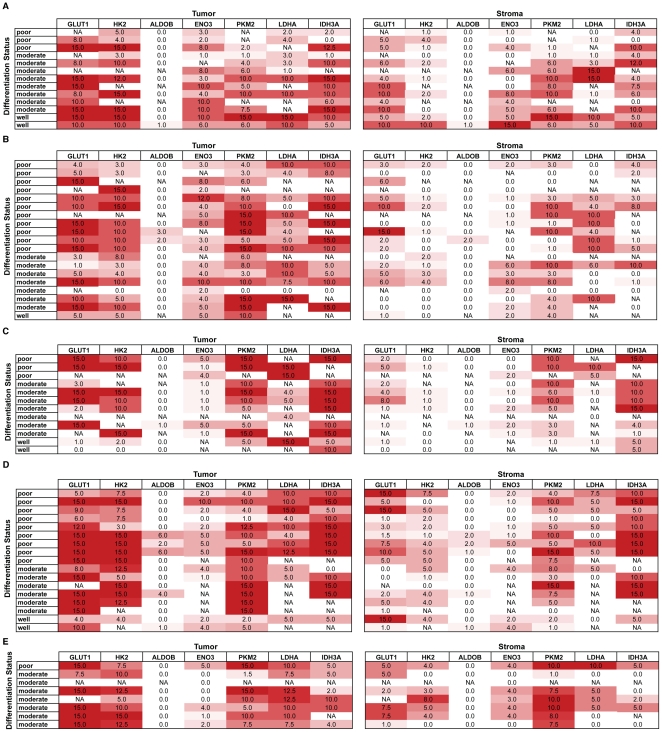
Semiquantitative evaulation of protein expression levels based on immunohistochemical findings. Tumor cell (left) and stromal (right) expression levels of GLUT1, HK2, ALDOB, ENO3, PKM2, LDHA and IDH3A in pancreatic tumor (A), and liver (B), lung (C), lymph node (D) and diaphragm (E) metastatic lesions were graded on a scale of 0–3. The staining intensities are tabularized and indicated with gradations of red color.

**Figure 4:**
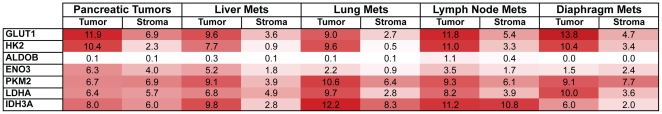
Differential protein expression levels by immunohistochemistry. Average tumor cell versus stromal cell expression intensities of GLUT1, HK2, ALDOB, ENO3, PKM2, LDHA and IDH3A in pancreatic tumor, and liver, lung, lymph node and diaphragm metastatic lesions are tabularized and indicated with gradations of red color.

Figure 3 presents the staining intensity scores for all the proteins in each specimen for tumor, as well as, stromal cells. Differential expression of proteins was observed between stromal and tumor cells for ENO3, GLUT1, HK2, IDH3A, LDHA and PKM2 (Table 2, Figure 4). However, the expression levels of ALDOB were not significantly different between stromal and tumor cells.

**Table 2:** Overall histoscore comparisons between the tumor and stromal cells.

Gene	Tumor^a^	Stromal^a^	*p* value^b^
GLUT1	15	4	1.72E-08
HK2	10	1	3.97E-14
ALDOB	0	0	0.560141
ENO3	4	1	1.11E-05
PKM2	10	6	0.000371
LDHA	10	5	0.001534
IDH3A	10	5	0.000913

a Median histoscores are presented for tumor and stromal cells.

b *p* value was calculated by Friedman's test on histoscore values.

Comparing the expression levels between the tissue sections from multiple patients, all pancreatic tumor specimens stained positive for GLUT1, HK2, ENO3, LDHA and PKM2. All liver metastatic lesions had positive stain intensities for GLUT1 and ENO3. All lung metastatic lesions had positive stain intensities for ENO3, PKM2, LDHA and IDH3A. All lymph node metastatic lesions had positive stain intensities for GLUT1, HK2, PKM2 and LDHA. All the diaphragm metastatic lesions had positive stain intensities for GLUT1, HK2, IDH3A, PKM2 and LDHA. In summary, GLUT1 was significantly expressed by all primary tumor and metastatic lesion specimens of pancreatic cancer, except lung metastatic lesions, and hence may serve as a good marker for pancreatic cancer overall. Moreover, staining for GLUT1 provided an median staining intensity score of 2 or above in all the primary and metastatic lesions (Figure 4), GLUT1 and IDH3A provided the largest median staining score of 15 in lung metastatic lesions. Furthermore, stromal cells from all pancreatic tumor sections and diaphragm metastatic legions stained positive for GLUT1. Stromal cells from all the pancreatic tumor, diaphragm and lung metastatic legions also stained positive for PKM2.

## Discussion

Pancreatic cancer displays alterations in cellular metabolism, which affects disease progression and represents a significant target to improve management of this disease [18], [19]. Several oncogenes and tumor suppressors have been implicated in altering tumor cell metabolism in order to facilitate tumor growth and metastasis [20], [21], [22]. Such alterations facilitate production of an abundant supply of key metabolic intermediates, which serve as essential ingredients for biosynthetic processes to sustain cell proliferation. The manner by which such alterations are involved in the progression of pancreatic cancer and if they differ between primary tumor and metastatic lesions at distant sites remain poorly understood. The studies presented here are the first to evaluate global metabolic alterations associated with metastasis to distant sites. We also evaluated the relative expression profiles of metabolic gene products between the tumor cells and the stromal cells to understand the nature of interactions in metabolic pathways between the tumor and stromal components of pancreatic adenocarcinomas.

Our studies confirm that the metabolic nature of pancreatic tumors is distinct from the normal pancreas. Most of the enzymes linked with aerobic glycolysis are expressed at low levels in the uninvolved pancreas, while they are significantly overexpressed in the primary tumors, as well as the metastatic lesions. However, we did not observe any significant changes in the expression of Krebs cycle pathway enzymes between the uninvolved pancreatic and tumor lesions. Although protein expression levels do not always correlate with the enzyme activities, expression levels of glucose transporters and metabolic enzymes have been correlated with higher flux of glucose through glycolytic pathways in pancreatic cancer [7], [15]. Hence, the increased expression of glycolytic machinery components suggests that pancreatic tumors might be more dependent on aerobic glycolysis as compared to the uninvolved pancreas.

We found that several genes associated with glucose uptake and glycolysis are upregulated in primary as well as metastatic tumors. GLUT1 is most abundantly overexpressed in primary as well as metastatic tumors. Consistent with the Warburg's hypothesis, we found that tumor cells demonstrate significant upregulation of glycolysis suggesting that most of the glucose that passes through the glycolytic pathway is eventually converted into lactate and secreted outside the tumor cells [2]. Since such a flux of glucose skips the entry of metabolites into TCA cycle, the energy production is significantly reduced [1]. To compensate for the growing need of energy and sustain the supply of metabolites for biosynthetic purposes, cells must allow for more glucose to be processed per unit time. Hence, upregulation of GLUT1 represents one such mechanism whereby increased entry of glucose ensures rapid proliferation and sustenance of biological processes critical for proliferation, metastasis, and adaptation and proliferation at the metastatic site [23]. Another enzyme, HKII, was significantly upregulated at primary as well as metastatic loci. Although the expression levels of HKII are not as prominently increased as GLUT1, this could be explained by the fact that HKII has a very low Km (0.1 mM); thus, it mostly functions at or near its maximal rate under physiological conditions [24]. Hence, even small increases in the levels of HKII will have significant impact on the overall levels of glucose turnover.

The metabolic gene expression profiles of distant metastases are predicted to be influenced by the cellular and stromal nature of the sites of metastases, which are distinct from that of the primary tumors. For example, IDH3A gene is underexpressed in liver compared to metastases in diaphragm, and expression levels in lungs and lymph nodes were even higher. IDH3A codes for the mitochondrial NAD^+^-specific isocitrate dehydrogenase α subunit, which can generate citrate, indirectly from pyruvate or glutamine, that can be converted by ACYL to Acetyl CoA, eventually contributing to membrane synthesis, by ACC and FAS catalyzed reactions. Although most of the glycolytic genes are significantly overexpressed in primary as well as metastatic tumors, the expression of enzymes that generate metabolites to feed into the biosynthetic pathways may be differentially altered between primary and metastatic sites. Overall, glycolytic genes show much higher variations in the expression levels than the genes involved in TCA cycle or PPP.

Our studies also demonstrate for the first time that the expression of glycolytic genes is significantly higher in the tumor cells than in the stromal cells of primary and metastatic pancreatic tumors. In fact, genes such as GLUT1 and HKII are upregulated over two-fold in the tumor cells as compared to the stromal cells. This supports the hypothesis that tumor cells preferentially consume glucose to generate lactate under aerobic conditions (aerobic glycolysis). The secreted lactate may serve as an energy source for stromal cells, which exhibit lower levels of glycolysis but more functional activity in the Krebs cycle. Lactate, which can reversibly be converted to pyruvate by pyruvate dehydrogenase, can then enter the Krebs cycle in the mitochondria to provide energy in the stromal cells. However, further studies will be needed to establish such metabolic relationships between tumor and stromal cells.

These studies also present GLUT1 as an overall metabolic marker for the pancreatic cancer cells, whereas, IDH3A may serve as an overall marker for the lung metastatic lesions of pancreatic adenocarcinoma. Identification of such markers might be significant in profiling the circulating tumor cells and predicting the patterns of metastasis in patients. Whether such expression patterns precede metastasis or are caused by the tumor microenvironment at the site of colonization, remains to be established.

Our studies compare metabolic gene expression both at the level of mRNA as well as protein. Several studies indicate that mRNA expression levels do not necessarily match with the protein levels in cancer [25]. At mRNA level, we did not observe any statistically significant changes for any genes in primary tumors and lung metastatic lesions as compared to the reference tissue. SLC2A1 (GLUT1) and PKM2 were the only genes that were significantly overexpressed at all the remaining sites (Table S1). However, at protein levels GLUT1, HK2, ENO3, PKM2, LDHA and IDH3 were all significantly overexpressed in contrast to normal pancreas. Our studies indicate that for primary and metastatic sites some gene expression profiles match at mRNA levels as well as protein level, while others demonstrate a discordant relationship. For example, GLUT1 and PKM2 have high expression levels at most sites in both mRNA and protein levels. HK2 demonstrates similar but less robust expression at mRNA level, while still significantly expressed at the protein level. LDHA demonstrate significant overexpression only for liver metastatic lesions at mRNA level, while it is significantly overexpressed at most sites at the protein level (Table S1). The discordant relationships indicate that the mRNA and protein levels may not be directly linked for most metabolic genes. Limited sample size for mRNA studies may be a factor for the modest changes observed. However, we believe that our studies will provide a scaffold for researchers investigating either mRNA or protein expression changes and correlating such alterations with diagnosis, progression and prognosis.

Very few studies exist that compare gene expression profiles for primary tumors as well as multiple metastatic lesions. Although not exactly comparable for the relative expression profiles as performed in our studies, we reanalyzed our mRNA expression profiles for primary tumors versus liver metastatic lesions in an attempt to validate our findings with an external dataset (GSE12630; Table S2). The comparison revealed that ALDOB, SLC2A2, PC and PCK1 mRNA levels were significantly higher in the liver metastatic lesions in comparison to the primary pancreatic tumors. According to our IHC results and published literature, expression levels of ALDOB, PC and PCK1, the latter two being gluconeogenic enzymes, tend to be higher in normal liver, a possible contaminant in the metastatic tissues utilized for RNA preparation and microarray [26]. However, the expression levels of SLC2A2 [27] tend to be significantly higher in tumor cells in contrast to the normal tissue. Hence, SLC2A2 was seemingly the only gene that was expressed significantly higher in liver metastatic lesions in comparison to the primary pancreatic tumors, in both the datasets.

The observed alterations in the expression of metabolic genes are predicted to result from modulations in oncogenic and tumor suppressor activities by the tumor cells. Pancreatic cancer cells carry activating mutations in several oncogenes and inactivating mutations in tumor suppressor. As 97% pancreatic cancer cells carry activating mutations in Kras, and at least 50% have mutated p53 tumor suppressor, it is predicted that such mutations and commensurate oncogenic events contribute to overexpression of the glycolytic genes. How such events lead to differential metabolic expression profiles in primary vs. metastatic lesions will be determined by future studies. Overall, our studies provide support for the concept that metabolism contributes to the aggressive nature of pancreatic tumors and their early metastases. Further studies will lead to development of metabolism-based biomarkers/therapeutics for the diagnosis/treatment of pancreatic cancer metastasis.

## Supporting Information

Table S1 **Gene expression ratios of metabolic genes in primary and metastatic tumors as compared to normal tissues.**
(XLS)

Table S2 **Comparison of metabolic gene expression profiles for primary tumor versus liver metastatic lesions and validation with an external dataset (GSE12630).**
(XLS)
